# University Student Perspectives of Entomophagy: Positive Attitudes Lead to Observability and Education Opportunities

**DOI:** 10.1093/jisesa/ieaa120

**Published:** 2020-10-24

**Authors:** Matthew Petersen, Olivia Olson, Sujaya Rao

**Affiliations:** Department of Entomology, University of Minnesota, St. Paul, MN

**Keywords:** entomophagy, edible insects, active learning, experiential learning, student opinion

## Abstract

Positive experiences with insect food items that highlight the benefits of insect production and reduce the novelty of entomophagy are needed. Toward this goal, we developed an experiential learning lesson plan that would provide a positive experience with entomophagy and associate key educational content related to insect food items. First, two cricket powder brownie taste-test surveys were conducted with groups of university students to evaluate attitudes relating to insects as food, sustainability of insect production, and nutritional content. Students displayed a taste preference for cricket flour brownies but could not consistently differentiate between brownie types, ranked environmental and nutritional benefits associated with insect food products over taste factors alone, and indicated a positive attitude toward purchasing insect products in the future. Willingness to try other insect products in the future was significantly greater for students with increased experience with consuming insect products. These results were then used to create an university lesson plan that will allows for future evaluation of student attitudes while increasing exposure to entomophagy and providing education on the positive aspects of insects as food production. Our work highlights the favorable attitude toward insect food products shown by university students and how positive perception of entomophagy increases with continued exposure to the practice.

Attention to insects as an alternative food source has increased over recent years and edible insects are predicted to occupy a larger share of the global food supply in the future (van Huis and Dunkel 2017). Globally, entomophagy has been embraced by many cultures and over 1,900 species of insects provide valuable nutrients to people’s diets (van Huis et al. 2013, Halloran et al. 2018, Kim et al. 2019). However, insect consumption is less common in many countries in the northern hemisphere, including the United States and most of Europe, where perceived social norms play an important role in dictating whether people will consume insects (Jensen and Lieberoth 2019). A major hurdle in getting people to consume insect products is getting them over the neophobia associated with the initial discomfort often felt when trying something new, such as consuming insect products (La Barbera et al. 2018, Schlup and Brunner 2018).

Education about insects, and insects as a potential food source, is believed to be critical to the acceptance of entomophagy in western regions (Hunts et al. 2019). As people become more familiar with entomophagy, they are more willing to accept it as a practice (Woolf et al. 2019). Integration of education related to the benefits of entomophagy with an ‘experience’ of eating insects or insect products in forms familiar to them may be a driver needed for large-scale adoption of entomophagy in the western world. Furthermore, the unique and novel experience of consuming insects can serve as a conduit for the delivery of material related to nutritional benefits and environmental issues associated with sustainable insect production.

Increased acceptance of insect products into the U.S. food stream offers clear environmental and nutritional advantages. Between 2012 and 2050, the global demand for animal proteins is expected to increase by 70–80% (Oonincx 2012). Insects present a food source rich in amino acids, mono- and polyunsaturated fatty acids, and several important micronutrients (Rumpold and Schlüter 2013). They also present a low-resource intensive source of food that requires less water, land space, and feed to be raised. Insect production also results in much less manure and up to 100 times fewer greenhouse gasses than traditional livestock (van Huis et al. 2013, van Huis et al. 2017, Halloran 2018). As an example, crickets are twice as efficient at converting feed to meat as chickens, at least 4 times as efficient as pigs, and 12 times more efficient than cattle (van Huis et al. 2013). Several trends have emerged indicating the main drivers for public acceptance of entomophagy reside in associating insects as food with the positive environmental, sustainable, and nutritional factors associated with consuming insects (Menozzi et al. 2017, Mancini et al. 2019b). Interestingly, there is also a perception of people who consume insect products as being health-conscious, environmentally friendly, and more knowledgeable than traditional meat consumers (Hartmann et al. 2018).

Outreach to the general public on the benefits of insects as food are needed, and a target populations for promotion should be undergraduates at universities and colleges. These students will face the challenges associated with an increasing population and a need for alternative sources of food. Fortunately, this demographic has shown a general willingness to consume insect products and may be viewed as early adopters of entomophagy (Mancini et al. 2019, Marquis et al. 2020). University students have also reported a positive association between entomophagy and environmental/sustainability issues (Menozzi and Sogari 2017, Sogari et al. 2017). Acceptance of entomophagy further increases when university students receive education related to subjects, such as entomology and food science (Sogari et al. 2017). The key to widespread adoption of entomophagy as an accepted practice may be rooted in integrated curricula that links the practice with underlying details of insect biology, food production, and sustainability issues surrounding insect production.

Presenting students with an integrated lesson plan that links food production with environmental issues can enhance student reception of content. Environmentally related sustainability course content can be more meaningful and impactful when it is integrated in an interdisciplinary way (Rowe 2007, Everett 2008, Jiusto et al. 2013) and can have a greater impact when the content can be conceptualized through a particular theme (Fisher and McAdams 2015). Combining student exposure to entomophagy with an associated lesson plan detailing the benefits of this practice can have a synergistic effect that will benefit education surrounding each subject.

To evaluate the potential of integrating entomophagy and a sustainability lesson plan, we instituted a procedure based on Hunts et al. (2019) that examined the gatekeepers limiting acceptance of insect food products into the U.S. food chain based on the theory of diffusion of innovation (Rogers 2003, see also Shelomi 2015). Specifically, we designed an active learning module that integrates a taste test (trialability) using chocolate cricket powder brownies (observability, compatibility) to introduce concepts of sustainability in food production systems (complexity, relative advantage). In a two-step study, we first administered insect taste-test surveys and an associated questionnaire to university student groups and students in an introductory entomology course as a way to evaluate their preference between conventional and cricket powder brownies, general perception of entomophagy, and how that perception was related to ideas of taste, environmental sustainability, and nutrition. This information was then used to develop an active learning lesson plan. The goals of this work are to evaluate current university student perceptions of entomophagy, and then develop a methodology for assessment of student attitudes in the future through an integrated lesson plan.

## Materials and Methods

### Cricket Powder Taste-Tests

We developed a taste-test survey to be administered to university students in two separate settings. The first was for student activity/interest groups (hereafter called campus survey) and the second was for students in an entomology course (hereafter called course survey). The willingness of the public to try new protein sources is generally low (Hartmann and Siegrist 2017); however, proper presentation of insect food products can greatly increase acceptance (Tan et al. 2016). While insect food products come in different preparations, the goal of this work was to examine student perception after consumption of the food product. We therefore wanted to decrease rejection based on presentation and instead present a food product that is common to the student demographic. For this reason, we chose to present the insect protein source in the form of a chocolate brownie that incorporated cricket powder. Brownies have been found to be an acceptable form for incorporating insect protein for student populations (Tan et al. 2016) and cricket powder is readily available for purchase by the public and can be easily incorporated into brownie recipes (Supp Table 1 [online only]).

Two batches of brownies were created: one using wheat flour and another using a mixture of cricket powder and wheat flour (Supp Table 1 [online only]). Brownies were baked using a commercial kitchen and then prepared into 2.5 cm^2^ samples. Brownie samples were presented to participants on two plates labeled as either A or B and students could randomly choose the order of sampling. Before students consumed either brownie, they were first informed of possible allergic reactions that may occur with consuming insect products and were advised to not participate in the survey if they had a shellfish allergy.

### Campus Survey

Student participants were gathered by contacting University of Minnesota (Twin Cities, MN) student groups from a wide range of disciplines (e.g., agriculture, business, environment, international, and athletics). A wide range of groups were contacted to gain perspectives from a relatively diverse group of student backgrounds and lifestyles. Student groups were contacted via e-mail, and the taste-tests and surveys were administered over several weeks during the student group’s regular meeting time in a campus classroom. Prior to the survey, students listened to a short slideshow presentation covering background information on the global practice of eating insects, environmental and nutritional benefits of the practice, and why crickets were chosen as the insect product for the taste-test.

During the survey, a cricket brownie sample (A) and a conventional/traditional brownie (B) were blindly distributed to each student. After the taste-test, students completed an online Qualtrics survey where they were asked questions about their demographics (e.g., age, gender, and area of residence), a series of questions relating to their opinions on eating insects and the brownies they just consumed, their preference between the two brownies, if they could guess which brownie contained cricket powder, and if they would consume and/or buy products made with cricket powder again (Table 1).

**Table 1.** Questions administered during the campus survey (Q) and course survey (C)

**Q1.**	**Have you ever intentionally eaten insects before?** Definitely not (5), Probably not (4), Not sure (3), Probably yes (2) Definitely yes (1).
**Q2.**	**Do you think brownie A or B had the cricket powder?** A, B, I have no clue!.
**Q3.**	**Which brownie did you like more?** A, B, I liked both equally, neither.
**Q4.**	**Would you willingly eat food made with cricket powder again?** Yes, Maybe, No.
**Q5.**	**How likely are you to try a different food item made with cricket powder?** Extremely unlikely (7), Moderately unlikely (6), Slightly unlikely (5), Neither likely nor unlikely (4), Slightly likely (3), Moderately likely (2), Extremely likely (1)
**Q6.**	**How likely would you be to purchase cricket powder for your own consumption (solely based on taste factors)?** Extremely unlikely (7), Moderately unlikely (6), Slightly unlikely (5), Neither likely nor unlikely (4), Slightly likely (3), Moderately likely (2), Extremely likely (1)
**Q7.**	**How likely would you be to purchase cricket powder for your own consumption (solely based on environmental factors)?** Extremely unlikely (7), Moderately unlikely (6), Slightly unlikely (5), Neither likely nor unlikely (4), Slightly likely (3), Moderately likely (2), Extremely likely (1)
**Q8.**	**How likely would you be to purchase cricket powder for your own consumption (solely based on nutritional factors)?** Extremely unlikely (7), Moderately unlikely (6), Slightly unlikely (5), Neither likely nor unlikely (4), Slightly likely (3), Moderately likely (2), Extremely likely (1)
**C1.**	**Pretest—On a scale of 1–10, what is your level of comfort with eating insects?** uncomfortable, very unlikely to eat insects (1), neutral (5), and very comfortable, very willing to eat insects (10).
**C2.**	**Do you think brownie A or B had the cricket powder?** A, B.
**C3.**	**Which brownie did you like more?** A, B.
**C4.**	**Post-Test—On a scale of 1–10, what is your level of comfort with eating insects?:** uncomfortable, very unlikely to eat insects (1), unlikely to eat insects (2.5), neutral (5), and comfortable (7.5), very willing to eat insects (10).

### Course Survey

A second taste-test was conducted in association with an introductory entomology course (ENT 1005 Insect Biology) at the University of Minnesota to both gauge the applicability of this survey to a classroom setting and to assist in the development of a lesson plan surrounding entomophagy.

The same brownie taste-test was used; however, no information about entomophagy was given prior to the test as was done in the campus survey. After agreeing to take part in the taste-test, students in the entomology course (*n* = 24) were first asked to rank their opinion about eating insects (Table 1). Students then randomly consumed an unlabeled cricket powder and convention brownie sample and were asked a series of oral follow-up questions by the course instructor relating to their opinions on eating insects and the brownies they just consumed (Table 1). Students in the course then presented the same survey to other university students outside of class (*n* = 37) as a way of increasing the sample size of the survey.

### Statistical Analysis

The student preferences for brownie type and guess of brownie type during both the campus and course surveys were evaluated using χ ^2^ goodness-of-fit test. The campus survey collected data on a Likerts 10-point (Course Survey) or 7-point scale (Campus Survey) and association of this ordinal data were evaluated using Spearman rank correlation. Differences in means for campus survey questions related to criteria influencing future purchases of insect products were evaluated using a Wilcoxon signed-ranks test. Finally, the course survey was developed to determine the mean change in student opinion related to eating insect products. This repeated-measures design was evaluated using a Wilcoxon signed-rank test. All statistical tests were conducted using R (R Core Team 2020).

### Lesson Plan Development

The results of the course and campus surveys were used to develop content for an active learning lesson plan that would integrate a taste-test survey with attributes associated with entomophagy (i.e., taste, nutritional content, and environmental information). Our goal was to develop a technique for increasing exposure about entomophagy among university students while establishing a methodology for the long-term monitoring of university student attitudes about entomophagy through an integrated course activity. Integrating a survey into a regularly occurring course would allow for structured data collection while simultaneously highlighting attributes associated with the use of insects as food items.

The results of the course and campus surveys were used to determine the willingness and reliability of students to take part in a taste-test survey, and thus the applicability of using this approach in a classroom setting and which associated attributes were most important to students. Insect food attributes that were seen as most important were chosen for inclusion in the lesson plan.

## Results

### Campus Survey

In total, 98 students took part in the survey. Participants had a mean age of 20 yr (<18 to 24 yr), identified as male (47.98%), female (51.02%), and nonbinary (1%), and came from city (21.43%), suburban (45.92%), and rural (32.65) areas. Nearly half of participants had probably (9.28%; *n* = 9) or definitely (38.14%; *n* = 37) eaten insect products before.

The majority of students (74.23%; *n* = 72) guessed incorrectly about which brownie contained the cricket powder, whereas 7.22% (*n* = 7) could not tell a difference (χ ^2^ = 77.25; df = 2, 97; *P* > 0.001) (Q2, see Table 2). The majority of students chose the brownie with cricket powder as the preferred brownie (71.13%; *n* = 69; χ ^2^ = 57.88; df = 1, 73; *P* > 0.001), whereas 20.62% (*n* = 20) enjoyed both equally and 4.12% (*n* = 4) did not like either (Q3).

**Table 2.** Student responses to brownie taste-tests

	Campus		Course	
Choice	Preferred	Guess	Preferred	Guess
Traditional	4 (4.12%)	72 (74.23%)	19 (39.58%)	14 (29.17%)
Cricket	69 (71.13%)	18 (18.56%)	29 (30.41%)	34 (70.83%)
Other	24 (24.74%)	7 (7.22%)	–	–
	*n* = 97	*n* = 97	*n* = 48	*n* = 48

Students in the campus survey preferred cricket powder brownies (χ ^2^ = 57.88; df = 1, 73; *P* > 0.001) and guessed they were traditional brownies (χ ^2^ = 77.25; df = 2, 97; *P* > 0.001). Course students had no preference (χ ^2^ =2.08; df =1, 48; *P* = 0.14) but correctly predicted those with cricket powder (χ ^2^ =8.33; df = 1, 48; *P* < 0.01).

A significantly larger number of participants were willing (69.39%; *n* = 68) or maybe willing (26.53%; *n* = 26) to eat, than not eat (4.08%; *n* = 4), foods made with cricket powder again (χ ^2^ = 67.47; df = 2, 98; *P* > 0.001) (Q4). The likelihood of students trying a different food item made with cricket powder in the future was associated with the likelihood of them having eaten insect products in the past (*r*_s_ = 0.31; *P* = 0.002) (Q5).

**Fig. 1.**
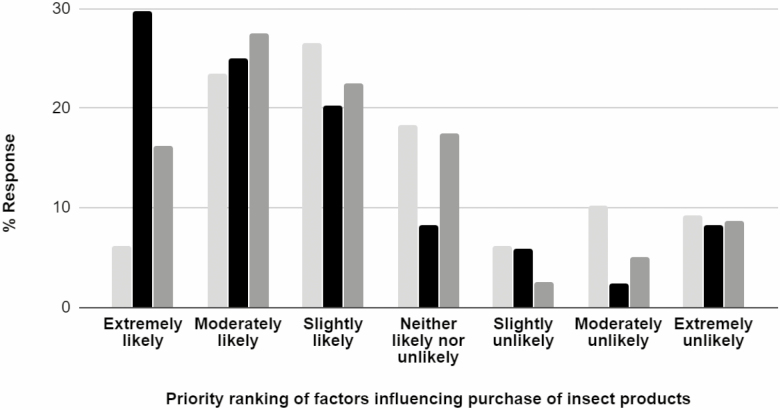
Campus student response to how likely they would be to purchase cricket powder for your own consumption based solely on taste (light gray), nutritional (gray), or environmental (black) factors)? Importance for taste factors was lower than environmental (*H* = 14.76; df = 1, 164 ; *P* < 0.001) and nutritional (*H* = 5.46; df = 1, 160; *P* = 0.019) factors. There was no significant difference between nutritional and environmental factors (*H* = 3.43; df = 1, 160; *P* = 0.06).

Likelihood of students purchasing cricket powder for consumption in the future (Q6–8) was lower for taste factors than for either environmental (*H* = 14.76; df = 1, 164; *P* < 0.001) and nutritional (*H* = 5.46; df = 1, 160; *P* = 0.019). There was no significant difference between nutritional and environmental factors (*H* = 3.43; df = 1, 160; *P* = 0.06). Overall, students were ‘slightly to moderately likely’ to purchase cricket powder in the future based on environmental factors, ‘neutral to slightly likely’ to do so based on nutritional factors, and ‘neutral to slightly likely’ to do so based on taste factors (Fig. 1).

### Course Survey

In total, 61 students were contacted to participate in the survey, with 13 declining to participate. Participants (*n* = 48) did not have a brownie preference (χ ^2^ =2.08; *P* = 0.14) (C2) but were able to predict which brownie contained the cricket powder (χ ^2^ =8.33; *P* < 0.01) (C3). Participants showed a significant change in opinion related to comfort level with consuming insects following the taste-test (*z* = −3.72; *n* = 27; *P* < 0.01), moving from a mean pretest response of slightly more positive than neutral (5.94; C1) and a posttest response of comfortable with the idea (6.90; C4).

### Lesson Plan Development

The results of the campus and course surveys indicated that students were accepting of eating insect food products in the form of cricket powder brownies. Students in the course survey that declined to take part in the survey did so based on being either vegan or vegetarian (*n* = 4) or because they were uninterested in consuming insects (*n* = 9). The high acceptance (79%) suggests that this approach should be useful in a class setting.

Students in the campus survey attributed acceptance of entomophagy to associated environmental and sustainability factors. We therefore developed a lesson-plan that highlights insect as food production as it relates to conventional beef/cattle production (Supp Material [online only]). This lesson-plan was aimed at evaluating student perceptions relating to entomophagy and creating an avenue to relate food production to environmental impact.

## Discussion

Education is critically important in developing the broader acceptance of entomophagy as it can change the perception from insects as pests, to insects as critical ecosystem components and sustainable resources. This is the first study to evaluate university student perceptions of entomophagy in order to develop methodology for education and future assessment of attitudes toward entomophagy. Here, we illustrate how the act of trying an insect food product increased the willingness of students to try and purchase another product in the future. Furthermore, there was a clear association between entomophagy and environmental and nutritional factors associated with insect products. These factors illustrate a developing trend for insect food products where consumer’s value information associated with food production (Menozzi and Sogari 2017, Sogari et al. 2017, Hunts et al. 2019) and nutrition (Hénault-Ethier et al. 2020) when determining their perception of the item itself. This process illustrates experiential learning, whereby students are active participants in the learning process. Here, students evaluated environmental and nutritional information to better comprehend their attitudes toward the insect food item and whether they would purchase a similar item in the future. Our results both contribute to a growing body of work that highlights the importance of supplementary information toward furthering the general acceptance of entomophagy over taste factors alone, and illustrate how entomophagy as an experiential learning activity can be used to promote student learning.

While our study focused on a single insect presentation, it suggests that taste alone is not a major determining factor for adoption of entomophagy among university students. Surveys suggested that students could tell a difference between the two brownies but did not associate the preference to flavor profile consistently. Campus survey students said that they thought cricket powder brownies should not taste good, expected the cricket powder to have a stronger flavor, or anticipated greater texture prevalence. Beyond personal preferences, positive associations (i.e., ‘tasty’) for insect products can be strongly impacted by the consumer’s background entomology knowledge (Hunts et al. 2019), by the influence of neophobia or perceived behavior control (Mancini et al. 2019). Clearly, the experienced taste profile for these students did not match the anticipated results. But given the contrasting results between surveys, with course students associating the preferred brownie with the insect product, taste alone did not seem to be a restricting barrier. Overcoming barriers linked to societal (Schlup and Brunner 2018, Jensen and Lieberoth 2019) or taste perception (Sogari et al. 2017) can play major roles toward eventual acceptance. While delivery of insect products through culturally or gastronomically acceptable products does increase willingness of the customer, our results suggest that interpretation of taste and preference related to cricket powder brownies can be highly subjective. Preference aside, it was clear that student perception about consuming insects was largely positive after consumption of the insect product itself and the ability to correctly predict the cricket powder brownie did not impact the final determination of whether they would eat or purchase insect products again.

Information beyond taste will be important for moving entomophagy beyond experience and toward an integrated component of student diet. Sustainability issues can be important drivers of consumer interest in economically developed countries (Lombardi et al. 2019) and willingness to purchase insect products in the future based on environmental or nutritional factors rather than taste alone has been well documented (Caparros Megido et al. 2014, Hartmann et al. 2017, Kostecka et al. 2017, Hénault-Ethier et al. 2020). The positive effects of intention illustrated here in the form of environmental and nutritional issues may counter the negative effects of perception in what an insect food product should taste like. Generally, the environmental impact of traditional animal protein production is not recognized by the general public (Hartmann and Siegrist 2017) and relaying and contrasting this information with the environmental impact of insect production appears to be a powerful approach. As edible insect production increases, it will be important to continuously evaluate these environmental assessments, as the impacts of different production systems can vary in their impact (Oonincx 2012).

These results help further a consensus surrounding the attitudes toward insect food choices and associated environmental and sustainability factors. Here, we ‘flipped the script’ by utilizing this body of knowledge to increase observability in entomophagy through education. We integrated an insect taste test into a lesson plan where students will find exposure that emphasizes the factors which increase adoption of new foods: increasing opportunities for exposure, expressing the benefits of the product, utilizing familiar flavors, and trust (Collins et al. 2019). Our entomophagy lesson consists of 1) a taste-test trial utilizing a culturally appropriate food presentation and 2) an integrated classroom plan that highlights environmental and sustainability benefits of insects as food (see Supp Entomophagy Lesson Plan [online only]).

This plan utilizes Western University student perception of insect foods as a way to create exposure for entomophagy through an experiential learning activity. Our goal was to maximize the potential for student acceptance through an appealing taste profile rather than attempting to fully examine student taste preferences. Therefore, different insect presentations (e.g., savory vs sweet, natural vs formulated) may present differing results (Hartmann et al. 2015, Tan et al. 2016, Hénault-Ethier et al. 2020) and modification of this lesson to fit the taste preferences of other regions may be necessary.

The second aspect of this lesson leads students through a statistical analysis of the survey data, and then tasks students with comparing conventional protein production with insect protein production. Experiential learning has been shown to greatly increase student performance in science, technology, engineering, and mathematics (STEM) courses (Freeman et al. 2014) and can serve as a platform for advancing conceptual understanding of statistical knowledge (Garfield and Ben-Zvi 2008). This lesson serves as an integrative approach that introduces students to entomophagy while introducing concepts of environmental sustainability and statistical analysis. It is appropriate as a standalone module or can be integrated into other subjects (e.g., Alternative Food Production).

The edible insect sector is expected to increase by over 47% between 2019 and 2026 (Ahuja and Mamtani 2020) and the acceptance of these products into people’s diets will, in part, be determined by type and number of positive exposures customers have with insect food products. Here, we provide a framework for the increased exposure of entomophagy to an accepting audience that values information about sustainable food production. If adoption is linked to sustainability, future success of this approach will depend on continual evaluation of the actual environmental impact of insect production. Particularly important will be the development of additional technological requirements needed for the successful large-scale processing of insects so they meet sustainability goals and can serve as a true protein alternative (Alexander et al. 2017, van der Weele et al. 2019) as well as lifecycle studies that measure and accurately report the impact of large-scale production are needed (Halloran et al. 2016).

Increased exposure to insects as food will increase familiarity within society. Future work will need to evaluate changing opinions toward insect foods over time, including the importance of multiple exposures, as well as defining sustainable insect production in terms of actual environmental impact. Familiarity acquired through experience is a powerful tool that can act to change the perceptions of the public. This work shows that exposure to cricket powder brownies along with information about sustainable insect production can increase the likelihood of Western University students consuming insect products in the future. The lesson plan presented here integrates these concepts into a single lesson plan as a way to increase exposure to entomophagy and integrate concepts of sustainability education.

## Supplementary Material

ieaa120_suppl_Supplementary_Material

## References

[CIT0001] Ahuja K, and Mamtani K. 2020 Edible insects market size by product (beetles, caterpillars, grasshoppers, bees, wasps, ants, scale insects & true bugs), by application (flour, protein bars, snacks), industry analysis report, regional outlook (US, Belgium, Netherlands, UK, France, China, Thailand, Vietnam, Brazil, Mexico), application potential, price trends, competitive market share & forecast2020 – 2026. Available from https://www.gminsights.com/industry-analysis/edible-insects-market. Accessed June 2020.

[CIT0002] Alexander P, Brown C, Arneth A, Dias C, Finnigan J, Moran D, and Rounsevell M D. 2017 Could consumption of insects, cultured meat or imitation meat reduce global agricultural land use? Glob. Food Sec. 15: 22–32

[CIT0041] Caparros Megido R, Sablon L, Geuens M, Brostaux Y, Alabi T, Blecker, D. Drugmand, E. Haubruge C, and Francis F. 2014 Edible insects acceptance by Belgian consumers: promising attitude for entomophagy development. J. Sen. Stud. 29(1): 14–20.

[CIT0003] Collins C M, Vaskou P, and Kountouris Y. 2019 Insect food products in the Western World: assessing the potential of a new ‘Green’ market. Ann. Entomol. Soc. Am. 112: 518–528. 31741488 10.1093/aesa/saz015 PMC6847481

[CIT0004] Everett J 2008 Sustainability in higher education: implications for the disciplines. Theor. Res. Soc. Educ. 6(2): 237–251.

[CIT0005] Fisher P B, and McAdams E. 2015 Gaps in sustainability education. Int. J. Sust. Higher Ed. 16(4): 407–423.

[CIT0006] Freeman S, Eddy S L, McDonough M, Smith M K, Okoroafor N, Jordt H, and Wenderoth M P. 2014 Active learning increases student performance in science, engineering, and mathematics. Proc. Natl. Acad. Sci. USA 111: 8410–8415. 24821756 10.1073/pnas.1319030111 PMC4060654

[CIT0007] Garfield J, and Ben-Zvi D. 2008 Developing students’ statistical reasoning: connecting research and teaching practice. Springer Science and Business Media, Berlin, Germany.

[CIT0008] Halloran A, Roos N, Eilenberg J, Cerutti A, and Bruun S. 2016 Life cycle assessment of edible insects for food protein: a review. Agron. Sustain. Dev. 36: 57. 32010238 10.1007/s13593-016-0392-8 PMC6961468

[CIT0009] Halloran A, Flore R, Vantomme P, and Roos N. 2018 Edible insects in sustainable food systems. Springer, London, United Kingdom.

[CIT0010] Hannah R 2020 Environmental impacts of food production. Available from https://ourworldindata.org/environmental-impacts-of-food. Accessed May 2020.

[CIT0040] Hartmann C, Ruby M B, Schmidt P, and Siegrist M. 2018 Brave, health-conscious, and environmentally friendly: Positive impressions of insect food product consumers. Food Qual. Prefer. 68: 64–71.

[CIT0011] Hartmann C, and Siegrist M. 2017 Consumer perception and behaviour regarding sustainable protein consumption: a systematic review. Trends Food Sci. Tech. 61: 11–25.

[CIT0012] Hartmann C, Shi J, Giusto A, and Siegrist M. 2015 The psychology of eating insects: a cross-cultural comparison between Germany and China. Food Qual. Prefer. 44: 148–156.

[CIT0013] Hénault-Ethier L, Marquis D, Dussault M, Deschamps M–H, and Vandenberg G. 2020 Entomophagy knowledge, behaviours and motivations: the case of French Quebeckers. J. Insects as Food Feed. 6(3): 245–259.

[CIT0014] van Huis A, and Dunkel F V. 2017 Edible insects: a neglected and promising food source, pp. 341–355. *In* Nadathur S R, Wanasundara J P D, and Scanlin L (eds.), Sustainable protein sources. Elsevier Inc., Amsterdam, The Netherlands.

[CIT0015] van Huis A, Van Itterbeeck J, Klunder H, Mertens E, Halloran A, Muir G, and Vantomme P. 2013 Edible insects: future prospects for food and feed security. Available from http://www.fao.org/docrep/018/i3253e/i3253e.pdf. Accessed June 2020.

[CIT0016] Hunts H J, Dunkel F V, Thienes M J, and Carnegie N B. 2019 Gatekeepers in the food industry: acceptability of edible insects. J. Insects Food Feed. 6(3): 1–14.

[CIT0017] Jensen N H, and Lieberoth A. 2019 We will eat disgusting foods together—evidence of the normative basis of Western entomophagy-disgust from an insect tasting. Food Qual. Prefer. 72: 109–115.

[CIT0018] Jiusto S, McCauley S, and Stephens J C. 2013 Integrating shared action learning into higher education for sustainability. J. Sust. Ed. 5 ISSN: 2151–7452.

[CIT0019] Kim T K, Yong H I, Kim Y B, Kim H W, and Choi Y S. 2019 Edible insects as a protein source: a review of public perception, processing technology, and research trends. Food Sci. Anim. Resour. 39: 521–540. 31508584 10.5851/kosfa.2019.e53 PMC6728817

[CIT0020] Kostecka J, Konieczna K, and Cunha L M. 2017 Evaluation of insect-based food acceptance by representatives of polish consumers in the context of natural resources processing retardation. J. Ecol. Eng. 18(2): 166–174.

[CIT0021] La Barbera F, Verneau F, Amato M, and Grunert K. 2018 Understanding Westerners’ disgust for the eating of insects: the role of food neophobia and implicit associations. Food Qual. Prefer. 64: 120–125.

[CIT0022] Lombardi A, Vecchio R, Borrello M, Caracciolo F, and Cembalo L. 2019 Willingness to pay for insect-based food: the role of information and carrier. Food Qual. Prefer. 72: 177–187.

[CIT0023] Mancini S, Moruzzo R, Riccioli F, and Paci G. 2019a European consumers’ readiness to adopt insects as food. A review. Food Res. Int. 122: 661–678. 31229126 10.1016/j.foodres.2019.01.041

[CIT0024] Mancini S, Sogari G, Menozzi D, Nuvoloni R, Torracca B, Moruzzo R, and Paci G. 2019b Factors predicting the intention of eating an insect-based product. Foods. 8(7): 270. 10.3390/foods8070270 PMC6678388 31331106

[CIT0025] Marquis D, Hénault-Ethier L, and LeBel J. 2020 Edible insect marketing in Western countries: wisely weighing the foodstuff, the foodie, and the foodscape. J. Insects as Food Feed. 6(4): 341–354.

[CIT0027] Menozzi D, Sogari G, Veneziani M, Simoni E, and Mora C. 2017 Eating novel foods: an application of the theory of planned behaviour to predict the consumption of an insect-based product. Food Qual. Prefer. 59: 27–34.

[CIT0028] Oonincx D G A B, and de Boer I J M. 2012 Environmental impact of the production of mealworms as a protein source for humans–a life cycle assessment. PLoS One 7(12): e51145. 23284661 10.1371/journal.pone.0051145 PMC3526541

[CIT0029] R Core Team. 2020 R: A language and environment for statistical computing. R Foundation for Statistical Computing, Vienna, Austria. Available from http://www.R-project.org/

[CIT0030] Rogers E M 2003 Diffusion of innovations, 5th ed. Free Press, New York.

[CIT0031] Rowe D 2007 Sustainability. Education for a sustainable future. Science. 317: 323–324. 17641184 10.1126/science.1143552

[CIT0032] Rumpold B A, and Schlüter O K. 2013 Nutritional composition and safety aspects of edible insects. Mol. Nutr. Food Res. 57: 802–823. 23471778 10.1002/mnfr.201200735

[CIT0033] Schlup Y, and Brunner T. 2018 Prospects for insects as food in Switzerland: a tobit regression. Food Qual. Prefer. 64: 37–46.

[CIT0034] Shelomi M 2015 Why we still don’t eat insects: assessing entomophagy promotion through a diffusion of innovations framework. Trends Food Sci. Technol. 45(2): 311–318.

[CIT0036] Sogari G, Menozzi D, and Mora C. 2017 Exploring young foodies’ knowledge and attitude regarding entomophagy: a qualitative study in Italy. Int. J. Gastron. Food Sci. 7: 16–19.

[CIT0037] Tan H S G, van den Berg E, and Stieger M. 2016 The influence of product preparation, familiarity and individual traits on the consumer acceptance of insects as food. Food Qual. Prefer. 52: 222–231.

[CIT0038] van der Weele C, Feindt P, van der Goot A J, van Mierlo B, and van Boekel M. 2019 Meat alternatives: an integrative comparison. Trends Food Sci. Technol. 88: 505–512

[CIT0039] Woolf E, Zhu Y, Emory K, Zhao L, and Liu C. 2019 Willingness to consume insect-containing foods: a survey in the United States. LWT – Food Sci. Technol. 102: 100–105.

