# Supplementary material for: University Student Perspectives of Entomophagy: Positive Attitudes Lead to Observability and Education Opportunities
Source: J Insect Sci. 2020 Oct 24;20(5):30. doi: 10.1093/jisesa/ieaa120 (PMC7585318; doi:10.1093/jisesa/ieaa120)
Supplement: ieaa120_suppl_Supplementary_Material [file ieaa120_suppl_supplementary_material.docx]

**Supplementary Material – Entomophagy Lesson Plan**

**Entomophagy: You want me to eat what?**

*-Course name / number-*

*-Date of lab-*

**INTENDED LEARNING OUTCOMES**

**On completion of the laboratory exercise, students will be able to:**

- **Describe how insects are used in food production**
- **Conduct a simple survey**
- **Quantify if a change in participant perception occurred after eating insects**
- **Relate consumer preferences to food production impacts**

Insects are the most abundant and diverse group of animals on the planet. They are found in nearly every terrestrial and aquatic habitat and play critical ecological functions in the environment. Simply put, our world would look very different without insects in it. But there is one place where you are unlikely to find insects…..on your dinner plate. Why is that, and should they be there? **Entomophagy**, or the practice of eating insects, is common to many cultures globally (Martin 2014). Currently, over 1,900 types of insects provide valuable nutrients to people’s diets (van Huis et al. 2013; Halloran et al. 2018).

Today we will explore the idea of insects as a food source. While many cultures incorporate insects into their diets, is there a reason why others do not? Is there a reason why increasing entomophagy makes sense? The goal of this exercise is to better understand entomophagy as a practice, and also why increased incorporation of insects into our food supply might make sense.

***Pre-Class Questions -*** Prior to class, read the attached article and answer the following questions.

[*Instructors note: there are several possible popular press articles that can be used here: How to Develop an Appetite for Insects: https://nyti.ms/2m08T1u; Why Aren’t We Eating More Insects? https://nyti.ms/2NorMH9; This low stakes introduction is about becoming familiar with the practice*]

*Q1: What types of insects are used in entomophagy?*

*Q2: Why aren’t insects typically consumed in North American diets?*

*Q3: Do you think insects should become a larger part of the American diet? Why or why not?*

***PART I – Insect Taste Test***

***Survey Procedure* -** This survey is divided into two components. First, you will take part in a blind taste test that compares your preference for cricket powder brownies against traditional chocolate brownies. Second, you will tabulate and analyze the collected data. The goals of this lab are to see if you can tell which brownie contains the cricket powder, and to determine if eating an insect can change people's perception about eating insects.

Two samples of brownies are prepared, one a traditional brownie and one with a portion of flour replaced with cricket powder (Table 1). The brownies have been cut into 2.5 cm^2^ portions. Your goal is to see if you can tell a difference between the two brownies. But first… **Question 1:** Rank your opinion about eating insects on a scale of 1 - 10, with 1 = uncomfortable, very unlikely to eat insects, 5 = neutral, and 10 = very comfortable, very willing to eat insects. Question 1_____.

On the plates labeled A and B are two types of brownies, one made with cricket powder and one traditional brownie. Important: If you have a shellfish allergy you may also be somewhat allergic to eating insects. Therefore you are advised not to take part in this taste test.

Now, eat each type of brownie (you can choose randomly). **Question 2:** Which brownie did you prefer, A or B? Question 2____. And...**Question 3:** Which brownie do you think contained cricket powder, A or B? Question 3____.

Now that you have consumed insects as food, take a minute and reconsider the original question (Question 1?) you were asked, **Question 4:** Rank your opinion about eating insects on a scale of 1 - 10, with 1 = uncomfortable, very unlikely to eat insects, 5 = neutral, and 10 = very comfortable, very willing to eat insects. Question 4_____.

You should now enter your information for questions 1-4 into the class spreadsheet.

[*Instructor note: Use an online database, like Google Sheets where the entire class can access the data*]

***Statistical Analysis -*** After eating the brownies, you know your individual preference, but individual data points such as this can only tell us so much about patterns or trends. Much like other types of scientific data, as researchers we look for trends and patterns by collecting multiple data points and then assessing change or difference. Now we will use the survey data for the class and two non-parametric statistical approaches to assess the goals of this lab - can the class tell which brownie contains the cricket powder and does eating an insect can change people's perception about eating insects in general?

*Procedure* - Your instructor will first give a short overview on the statistics we will be using in this lab. You can take notes here:

[*Instructor note: I find it better to give a short lecture on the two approaches rather than having the descriptions already given*]

We will use a simple online calculator to analyze our data. First go to https://www.socscistatistics.com/tests/ and navigate to the Chi-squared Goodness of Fit test. Enter A and B as out groups (these are the brownie types). Click the Next button. Under Observed, enter the actual counts for the votes for which brownie was preferred. Under Expected, sum the counts for A and B and divide that number by 2. Put this number in the Expected cell for both A and B. Then Calculate the Chi-square (*X*^2^) value.

*X*^2^ value:_______________ p-value:______________________

*Q3: Interpret the results of this analysis. Is there a difference in preference between brownie A and B? What does this suggest?*

Now repeat this procedure for the data associated with the guess of which brownie contained the cricket powder.

*X*^2^ value:_______________ p-value:______________________

*Q4: Interpret the results of this analysis. Is there a difference in guesses between brownie A and B? What does this suggest?*

Finally, can eating an insect product change people's opinion about eating insects in general? Navigate to the Wilcoxon Signed-Ranks Calculator. Copy and paste into Treatment 1 the data related to Question 1. Then, copy and paste into Treatment 2 the data from Question 4. Remember, these are paired data so make sure not to change the order of answers when copying and pasting. Then Calculate.

p-value:______________________

*Q4: Did eating the cricket brownie change the perception about eating insects?*

[*Instructor note: For the inclusion of the statistical analysis of collected data, sample sizes need to be sufficiently large. To test for statistical differences in the data, a minimum sample size of 25 students is needed. Participant numbers can be increased by modifying this assignment to be a survey of students outside of the course. The student preference of brownie type (Question 2) and guess of brownie type (Questions 3) are evaluated using a Chi-square goodness-of-fit test. The change in opinion after eating the cricket brownie (Questions 1, 4) is evaluated using a Wilcoxon signed-ranks test. Both statistical tests have the advantage of being easily computed using Microsoft Excel, Google Sheets, or through several online calculators (e.g., https://www.socscistatistics.com/tests/). More advanced lessons may wish to use advanced statistical packages (e.g., SAS, R)*]

***PART II – Beyond Taste: Insects as Sustainable Food Products***

Insects might be an acceptable addition to our diets. But could insects as food production ever become widely accepted by the general public? For this to happen, two broad criteria would need to be met. First, the general public would need to accept the product (which you may or may not have proven in PART I). Second, there has to be a reason why the product is better (or different) than products already in existence. We will explore this second idea a bit more in terms of sustainability.

**Sustainable** food production can be described as a system that provides food to meet current, and future, demand while maintaining healthy ecosystems and ensuring minimal negative impact to the environment. Ultimately we are interested in the environmental impact of the food production, and the degree to which the food is nutritious and meets food demand. For this assignment we will focus on the latter, and describe the environmental impact of insects as sources of protein by comparing insects to one of the most common form of food protein, cattle and beef production.

Part I of this exercise was based on personal preferences. However, we used statistical analysis to quantify the differences in opinion. Similarly, here we want to quantify differences in production systems by providing measurable values and actual data. In the realm of science we deal in the currency of data and evidence, and as you answer the following questions you should similarly provide data and evidence to support your claims.

Your instructor will assign you to groups. Your assignment is to relate the environmental impact of insects produced for food to that of conventional beef/cattle production.

**Conventional Agriculture** – First, let us examine the environmental impacts of food production. As a group, use the data on “Environmental impacts of food production” (Ritchie, 2020; https://ourworldindata.org/environmental-impacts-of-food) to answer questions Q5-Q6. The best way to address these questions is to first review the information on the site as a group to identify the categories of impact. Then, assign individual group members to first describe individual categories of environmental impact (Q5), and then specifically address how these impacts relate to beef/cattle production (Q6). Your final answer should incorporate information from each group member.

*Q5: Identify and describe the categories of environmental impacts caused by conventional food production.*

*Q6: Conventional beef/cattle production can be described as one of the least sustainable food production systems. Describe why this is so.*

**Insect Production** – You may have noticed that insect production was not included in the data on this site. Insects in food production is still a fairly new endeavor, and quantifiable data is not fully available yet. To get an idea of how sustainable insect production is, we will use the paper “Comparing Environmental Impacts from Insects for Feed and Food as an Alternative to Animal Production” (Halloran et al. 2018). As a group, use this paper to answer the following questions. Again, divide the group to address topics before coming together to develop a single collaborative answer.

*Q7: Describe how insect production relates to the categories of environmental impacts you identified in* Q5*. Can insect production for food be described as a more or less sustainable process than beef/cattle production?*

*Q8: What limitations exist that do not allow us to fully quantify the environmental impact of insect production?*

[*Instructor note: the assignment can be used as a group or individual assignment, or as a homework assignment depending on time allowed in class. The important aspect of the assignment is that students interpret the results of the statistical analysis, and then develop an argument based on the environmental or nutritional benefits of entomophagy. After they develop those arguments you can discuss them as a class. You may also want to then ask them to rank their opinion relating to eating insects - Question 4*]

[*Instructor note: I have highlighted two papers/sources that can be used for the purpose of this assignment, however there are many additional papers that can be used. The issue of The Journal of Insect Science – Insects as Food and Feed: if you can’t beat them, eat them! is a great source of information*]

| **Supp. Table S1. Cricket brownie recipe.** For the ‘traditional’ brownie recipe substitute the cricket powder with whole wheat flour. |
| --- |
| ***CRICKET BROWNIES***  *2 oz unsweetened chocolate (= 8 ¼ oz pieces)*  *1/2 Cup butter (2 half sticks)*  *3/4 Cup sugar*  *2 eggs*  *1/2 Cup cricket baking powder*  *1/8 tsp salt*  *1/2 tsp vanilla*     1. *Melt unsweetened chocolate with the butter in microwave, stirring every 30 secs until melted; cool* 2. *Mix eggs, sugar, and vanilla until well blended, combine with chocolate mixture* 3. *Fold in flour and salt* 4. *Pour in greased pan, bake at 350°F in an 8 inch square pan for 15-20 mins. Remove from oven when brownies are still soft on top. Cool and cut into squares* |

**References:**

**Halloran, A., Flore, R., Vantomme, P., Roos, N. 2018.** Edible insects in sustainable food

systems*.* Springer Nature, Switzerland.

**Martin, D. 2014.** Edible: An adventure into the world of eating insects and the last great hope

to save the planet*.* Houghton Mifflin Harcourt Publishing, New York, NY, USA.

**Hannah, R. 2020.** Environmental impacts of food production. (OurWorldInData.org; https://ourworldindata.org/environmental-impacts-of-food).

**van Huis, A., Van Itterbeeck, J., Klunder, H., Mertens, E., Halloran, A., Muir, G., Vantomme, P. 2013.** Edible insects: Future prospects for food and feed security*.* (http://www.fao.org/docrep/018/i3253e/i3253e.pdf **).**
